# Impact of HPV Vaccination on Juvenile Laryngeal Papillomas and Conjunctival Papillomas in Denmark: Incidence and Analysis of HPV Subtypes in Relation to Vaccination

**DOI:** 10.1111/apm.70240

**Published:** 2026-08-19

**Authors:** Mathilde Aakilde, Joachim Hansen, Ida‐Marie Jacobsen, Simone Kloch Bendtsen, Giedrius Lelkaitis, Steffen Heegaard, Christian von Buchwald

**Affiliations:** ^1^ Department of Otorhinolaryngology, Head and Neck Surgery & Audiology Copenhagen University Hospital ‐ Rigshospitalet Copenhagen Denmark; ^2^ Department of Pathology Copenhagen University Hospital ‐ Rigshospitalet Copenhagen Denmark; ^3^ Department of Ophthalmology Copenhagen University Hospital ‐ Rigshospitalet Copenhagen Denmark

**Keywords:** conjunctival papilloma, HPV vaccine, JoRRP, laryngeal papilloma

## Abstract

Papillomas, predominantly caused by low‐risk Human papillomavirus (HPV), can affect the respiratory tract and conjunctiva. This nationwide Danish registry study assesses the HPV vaccination programme's effectiveness in preventing juvenile laryngeal and conjunctival papillomas. Incidence rates were calculated using national registry data from 2005/2008 to 2024. In the Danish Pathology Registry, 33 juvenile laryngeal and 134 conjunctival papilloma patients were identified using SNOMED codes. HPV subtyping was performed in‐house for laryngeal biopsies lacking prior data, yielding results for 27 patients. HPV‐6 or ‐11 was identified in 74.1% of cases. Vaccination status was retrieved for conjunctival patients and for mothers of laryngeal patients. The incidence rate of juvenile laryngeal papillomas was 0.190 per 100,000 (2005–2013) and 0.107 (2014–2024) (*p* = 0.145). No mothers of laryngeal patients had received HPV vaccination before delivery. Conjunctival papilloma incidence declined from 2008 to 2024, with an annual percent change of −5.3% (*p* = 0.002). A total of 22 conjunctival patients were vaccinated before first biopsy, with a median interval of 5 years and 8 months between first dose and biopsy. In Denmark, juvenile laryngeal papilloma incidence remained low and occurred only in children of unvaccinated mothers, while conjunctival papillomas showed a significant decline consistent with a protective effect of the HPV vaccination programme.

## 1 Introduction

Papillomas are benign epithelial tumours typically caused by low‐risk Human papillomavirus (HPV) [1]. In the respiratory tract, repeated growth of papillomas is known as recurrent respiratory papillomatosis (RRP). RRP is typically restricted to the larynx. Still, recurrent involvement of the nasopharynx and distal airways is observed [2]. The juvenile‐onset form of RRP (JoRRP) typically presents between age 3 and 4 years and often requires multiple surgeries to maintain an open airway and voice function. In severe cases, distal spread, tracheostomy, and fatal outcomes may occur [3]. JoRRP is believed to be acquired through vertical transmission of HPV, predominantly types 6 and 11, during delivery via an infected genital tract or through intrauterine transmission [2]. A major risk factor for the development of JoRRP is maternal genital warts during pregnancy and delivery [3]. Genital warts are also mostly caused by HPV 6 and 11 and are sexually transmitted [1]. Other HPV subtypes, including 16, 18, 31, and 33, are also associated with RRP but with a lower prevalence [2]. Malignant transformation occurs in less than 1% of JoRRP cases [2].

Conjunctival papillomas are, similar to laryngeal papillomas, also predominantly associated with HPV types 6 and 11 and can be transmitted during birth [4, 5]. Another mechanism of infection in both children and adults is believed to be via ocular contact with contaminated hands, as HPV DNA has been detected on the fingers of patients with genital HPV infection [4, 6]. Conjunctival papillomas are more common in men, most frequently occurring between 21 and 40 years of age, and may cause ocular discomfort, cosmetic concerns, or impaired visual acuity, including amblyopia in children, when extensive [5].

Implementation of the HPV vaccine has introduced the possibility of preventing conjunctival papillomas and JoRRP, as it offers protection against several HPV types including 6, 11, 16, and 18 [3]. Studying these two rare diseases together allows assessment of both direct and delayed vaccine effects, as conjunctival papilloma reflects direct protection in vaccinated individuals, whereas JoRRP reflects indirect effects through maternal vaccination.

The HPV vaccine was implemented in the Danish childhood vaccination programme for 12‐year‐old girls in January 2009 and was preceded and followed by several catch‐up programmes, including one for women aged up to 27 years in 2012 [7]. From 2019, boys born after July 2007 were also included in the programme along with additional catch‐up programmes for selected male cohorts [8, 9]. Since implementation, three different HPV vaccines have been used, each covering different HPV types [7]. Currently, girls and boys aged 12–17 years are offered the vaccine, with a two‐dose schedule for those initiating vaccination before the age of 15 years [10].

The average coverage for fully vaccinated Danish girls born from 1993 to 2010 is 77.3% [11]. The coverage for boys born between 2006 and 2010 is 73.8% [12]. The prevalence of vaccine‐targeted HPV types has declined among young women following the implementation of HPV vaccination, contributing to the prevention of diseases such as cervical cancer [3]. No studies have examined conjunctival papilloma incidence after vaccine introduction, and while JoRRP incidence has declined in the United States and Australia [13], the association between maternal vaccination and HPV subtypes in children's laryngeal papillomas remains unstudied.

### 1.1 Aim and Hypothesis

This study aims to investigate whether the introduction of the HPV vaccine into the Danish childhood vaccination programme in 2009 has led to a reduction in the incidence rates of conjunctival papillomas and juvenile laryngeal papillomas in Denmark. We hypothesise that the incidence rates of both papilloma types have declined following HPV vaccine implementation. Furthermore, we expect that post‐2014 cases of juvenile laryngeal papillomas predominantly occur in children born to unvaccinated mothers. This hypothesis is based on the 2012 national catch‐up programme, which offered HPV vaccination to women up to 27 years of age. A latency period was required for these vaccinated women to conceive and give birth, resulting in an expected protective effect of maternal immunisation from around 2014 onwards.

## 2 Materials and Methods

This is a nationwide Danish retrospective registry study. A dataset from the Danish National Pathology Registry (Patobank) was extracted using SNOMED codes. Data comprised biopsies of laryngeal and conjunctival papillomas collected between 2005 and 2024 from all regions of Denmark (excluding December 2024). Each biopsy was linked to a personal identification number (CPR number) and date of the biopsy.

### 2.1 Outcomes

The primary outcome was to evaluate the incidence rates of juvenile laryngeal papillomas and conjunctival papillomas after the introduction of the HPV vaccine in the Danish childhood vaccination programme. Secondary outcomes were:

- Evaluation of maternal HPV vaccination and incidence of laryngeal papillomas in their children
- Evaluation of maternal HPV vaccine and the association with the HPV subtype found in laryngeal biopsies from their children
- Assessment of HPV vaccination status in patients with conjunctival papillomas

### 2.2 Identification and Data Collection for Juvenile Laryngeal Papillomas

We identified patients who had biopsies taken from laryngeal papillomas before the age of 18 years between 2005 and 2024 in Denmark. The 2005–2024 study period was selected to ensure sufficient pre‐vaccine baseline data for evaluating incidence trends. Only the first recorded biopsy for each patient was included, and only biopsies from papillomas located in the larynx were included. As we did not differentiate between recurrent (JoRRP) and non‐recurrent cases of laryngeal papillomas in juvenile patients, the condition is referred to collectively as juvenile laryngeal papillomas throughout this study. For patients where HPV subtyping had not been previously performed, stored biopsy samples were analysed for HPV subtypes at the Department of Pathology, Copenhagen University Hospital—Rigshospitalet. Due to feasibility, biopsies were collected only from the biobanks in the Capital region and Zealand Region of Denmark, covering ~47% of the Danish population. Additionally, the HPV vaccination status of the patients' mothers was acquired to evaluate the potential preventive effect of the vaccine.

### 2.3 Identification and Data Collection for Conjunctival Papillomas

We identified patients with conjunctival papillomas aged 0–40 years, who had biopsies taken from 2008 to 2024 in Denmark. We ended data collection at age 39 years due to the lack of HPV vaccination in the age groups beyond this age. The HPV vaccination status of each patient was obtained. HPV subtyping was not conducted for conjunctival papillomas.

### 2.4 Data Sources

The Danish Pathology Registry is a nationwide database containing reports from all pathology examinations conducted by the Danish pathology departments. Vaccination status was obtained from the Danish Health Data Authority, including HPV vaccine type and dates of each administered dose. For juvenile laryngeal papilloma cases, this enabled comparison with offspring birth dates to determine whether maternal vaccination occurred prior to pregnancy and, for conjunctival papilloma cases, whether individuals had been vaccinated prior to their first biopsy. Background population data for statistical analyses were obtained from Statistics Denmark and were reported as population counts on January 1 for each study year.

### 2.5 Biopsy Analysis

The biopsies were archived as formalin‐fixed, paraffin‐embedded tissue samples in existing clinical biobanks. The biopsies were subtyped using VisionArray HPV Chip 1.0—a PCR‐based method that tests for 41 HPV types simultaneously. All samples were confirmed to contain papillomatous tissue through haematoxylin and eosin (H&E) staining by our pathologist (GL).

### 2.6 Statistical Analysis

All statistical analyses were conducted using RStudio software (version 4.4.1). A *p* value < 0.05 was considered statistically significant.

Incidence rates were calculated by dividing the number of new cases by the corresponding background population for each year within the defined time periods, using the background population aged < 18 years for the laryngeal cases and the background population aged < 40 years for the conjunctival cases. Rates are presented per 100,000 person‐years.

#### 2.6.1 Statistics Regarding Juvenile Laryngeal Papillomas

To assess the potential preventive effect of maternal HPV vaccination, patients were grouped into two periods (2005–2013 and 2014–2024) based on expected HPV vaccine impact. Incidence rates were calculated for each period. The 2014 cut‐off reflects the introduction of catch‐up HPV vaccination for women up to 27 years in 2012. Incidence rates with 95% confidence intervals were estimated using the Poisson distribution, and differences between periods were assessed using an exact binomial test in R. Post hoc power was estimated using the Poisson distribution.

#### 2.6.2 Statistics Regarding Conjunctival Papillomas

To investigate whether there was a statistically significant decline in incidence over time, we applied a Poisson regression model with a log‐link function. The logarithm of the annual population was included as an offset, thereby modelling incidence rates rather than raw case counts. Year was entered as a continuous predictor, allowing correct testing of a temporal trend. From the estimated regression coefficient for year, we derived the annual percent change (APC) and corresponding rate ratios (RR) with 95% confidence intervals.

## 3 Results

### 3.1 Juvenile Laryngeal Papillomas

**FIGURE 1:**
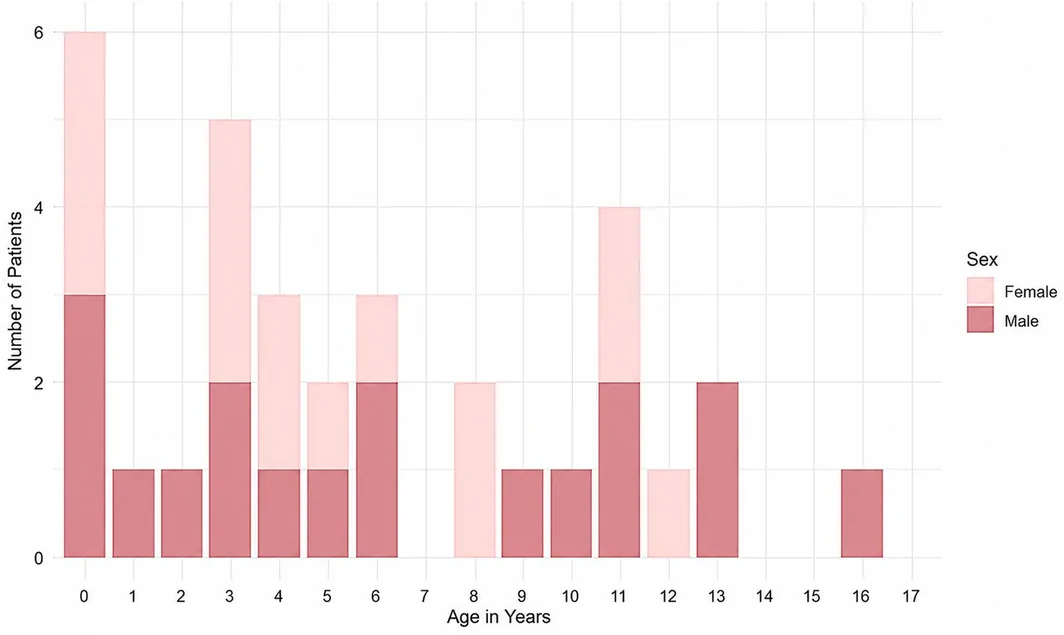
Distribution of patients with laryngeal papillomas according to age and sex from 2005 to 2024. It shows 15 females and 18 males with laryngeal papillomas. The majority of patients are aged 7 years or younger. The highest number of patients underwent their first biopsy at the age of 0 years. Median age is 5 years.

**FIGURE 2:**
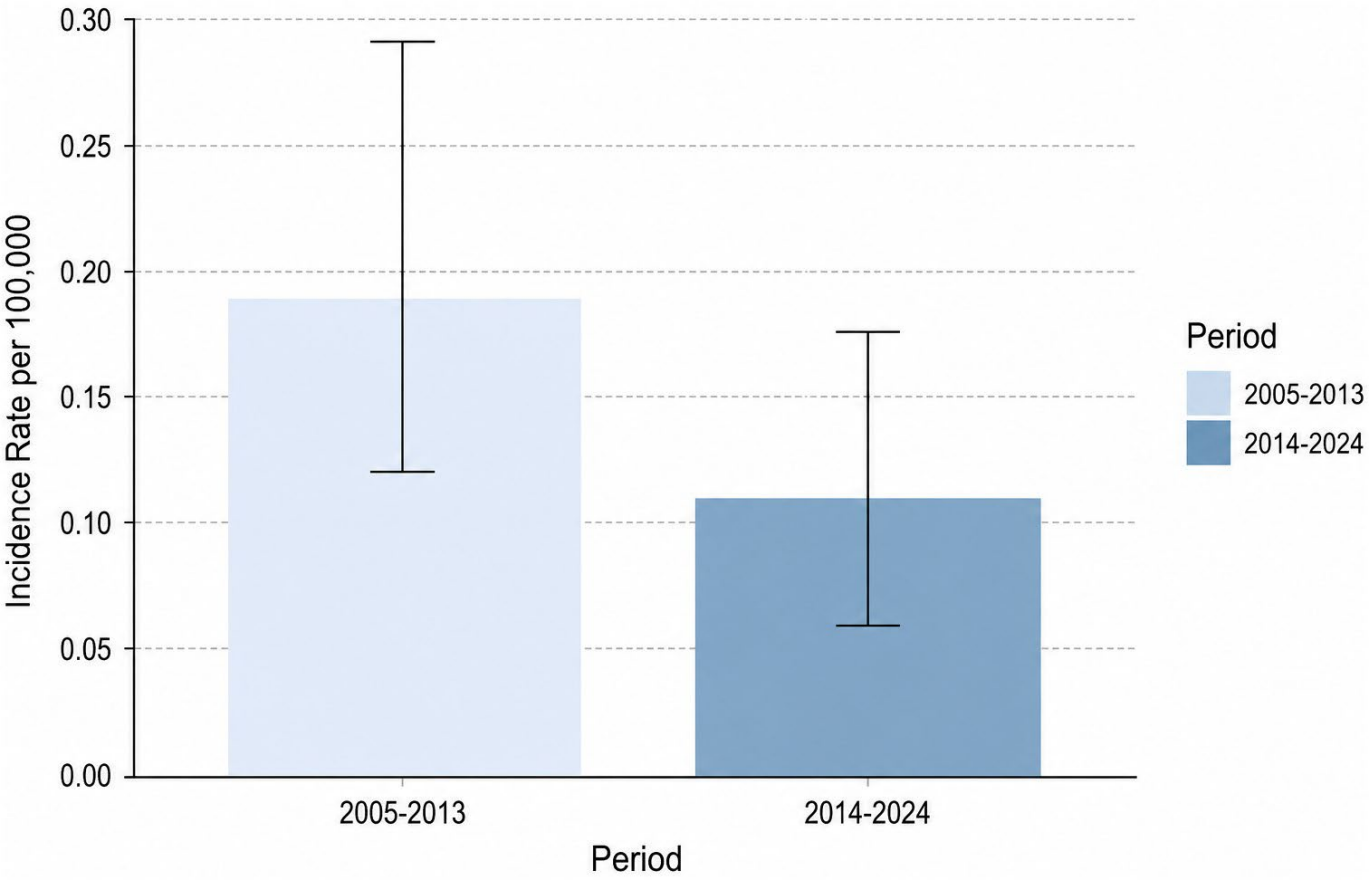
Incidence rates of juvenile laryngeal papillomas before (2005–2013) and after (2014–2024) expected effect of vaccination. Incidence rates of juvenile laryngeal papillomas were 0.190 per 100,000 (2005–2013) and 0.107 (2014–2024) (*p* = 0.145). There was no significant difference in incidence rates between 2005–2013 and 2014–2024.

We included 33 patients, 45.5% (15) females and 54.5% (18) males. Mean age at first biopsy was 5.8 years and median age was 5 years (age interval 0–16 years) (Figure 1). The incidence rate for the period 2005–2013 was 0.190 per 100,000 (95% CI: 0.117–0.296) and the incidence rate for the period 2014–2024 was 0.107 per 100,000 (95% CI: 0.057–0.185) (*p* = 0.145). Bar plot visualisation highlights a lower incidence rate in the later period (Figure 2). Post hoc power analysis showed a statistical power of 18% to detect a difference in incidence between the two time periods.

**FIGURE 3:**
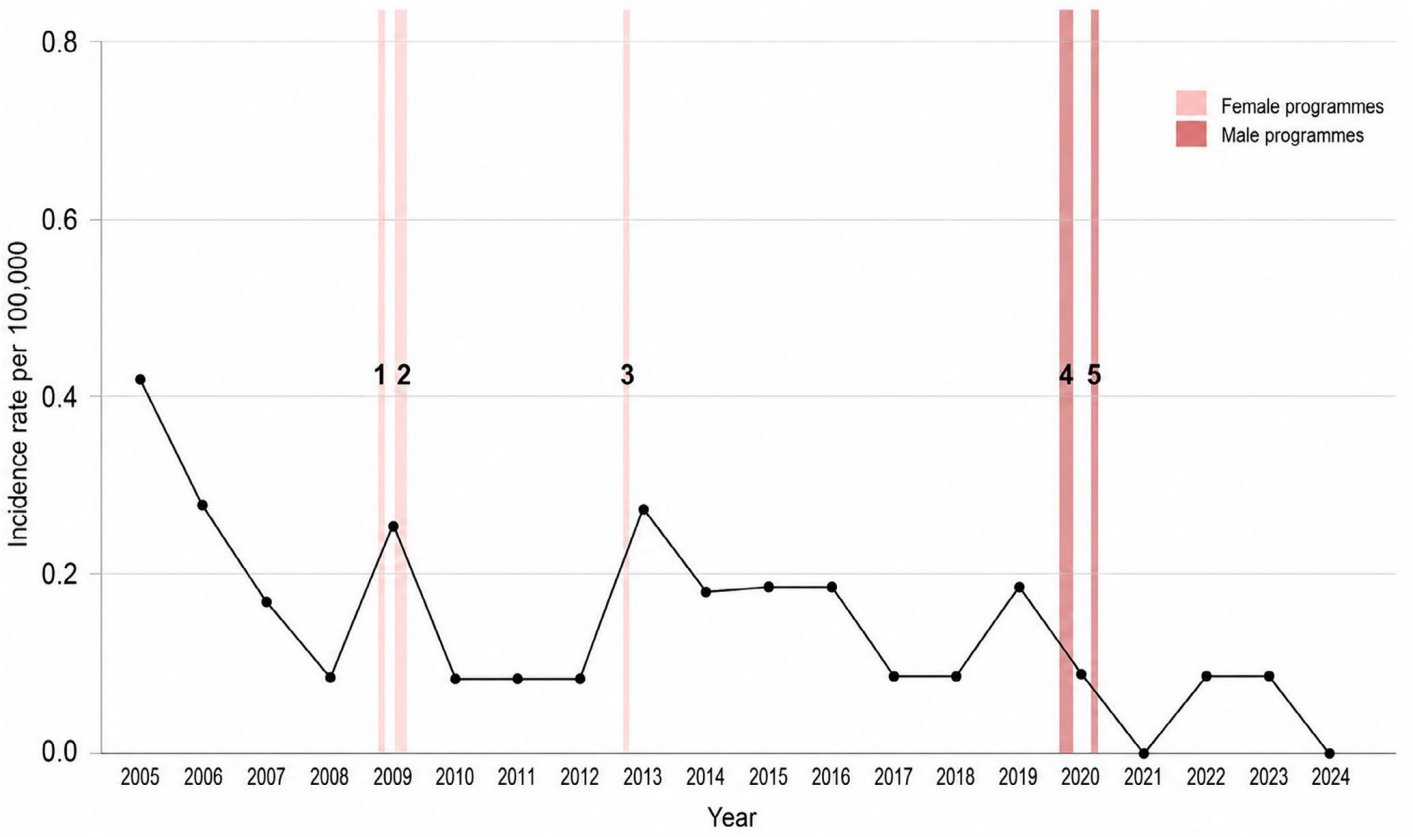
Yearly incidence rates of juvenile laryngeal papillomas and implementation of HPV vaccines. It shows declining incidence in juvenile laryngeal papillomas from 2005 to 2024. From 2020 onward, incidence rates have remained between 0 and 0.1 per 100,000. Vertical lines: Light pink: Female HPV vaccination, Dark pink: Male HPV vaccination, Thick lines: Official national implementation. 1: First catch up programme, girls aged 13–15 years. 2: Implementation of national HPV vaccination for 12‐year‐old girls. 3: Second catch up programme, women aged up to 27 years. 4: Implementation of national HPV vaccination for boys born after 1/7–2007.5: Two catch up programmes. One included boys born from 1/1–2006 to 30/6‐2007, the other included young men born between 1/1‐1994 and 31/12‐2003 who were attracted to other men.

A graphical representation of the incidence rates over time, with vertical reference lines indicating the introduction of the HPV vaccination for girls and boys including catch up programmes, demonstrates a fluctuating pattern with a decreasing trend (Figure 3).

HPV subtype status—including cases in which HPV was not detected—was available for 27 of the 33 patients. A total of 17 patients had HPV subtyping conducted prior to this study, while an additional 10 patients were analysed as part of this study using the VisionArray HPV Chip 1.0. Among these 10 newly analysed samples, 5 were HPV‐negative despite a valid cellular control signal.

**FIGURE 4:**
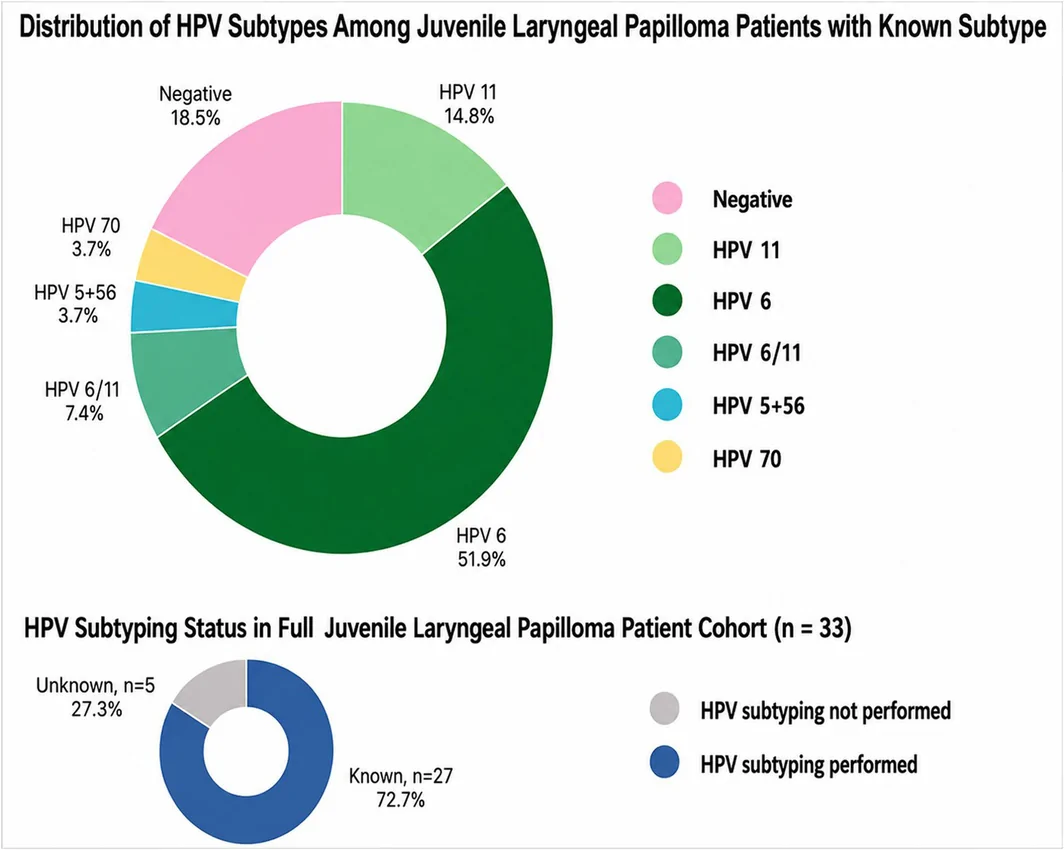
HPV subtypes in juvenile layngeal papilloma biopsies. HPV 6 was detected in more than 50% of the juvenile laryngeal papilloma biopsies. 18.5% were negative for HPV.

Of the 27 patients, 14.8% (*n* = 4) had HPV 11, 51.9% (*n* = 14) had HPV 6, 7.4% (*n* = 2) were classified as 6/11. In total, 74.1% of the 27 patients had HPV 6 or 11. Further, 3.7% (*n* = 1) had combination HPV subtype 6 + 56. This patient did not show signs of malignancy pathologically. 3.7% (*n* = 1) had HPV 70 and in 18.5% of cases (*n* = 5), HPV was not found (Figure 4).

Mothers of 31 children were identified and none of these had received HPV vaccination prior to delivery of the children included in our study.

### 3.2 Conjunctival Papillomas

**FIGURE 5:**
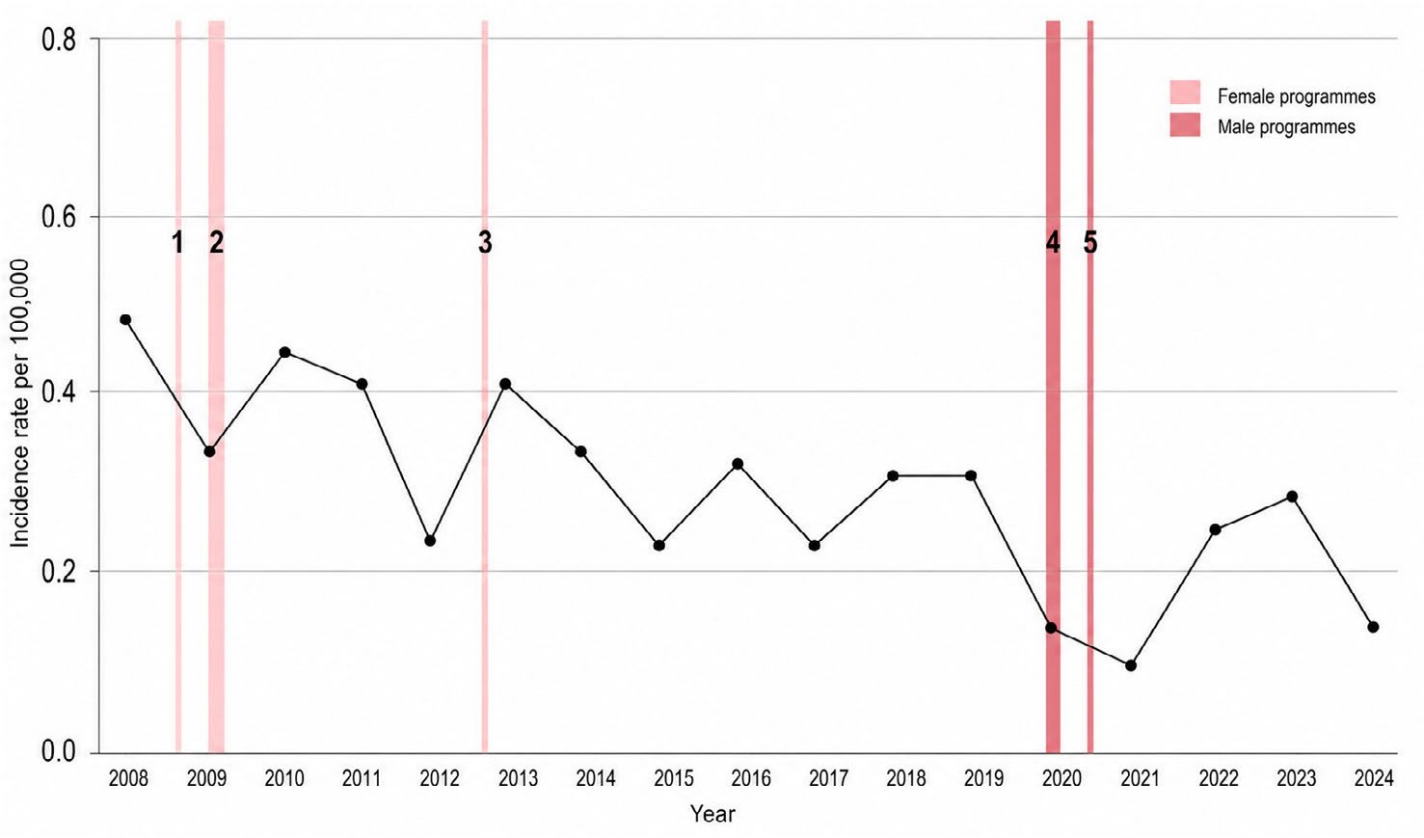
Yearly incidence rates of conjunctival papillomas and implementation of HPV vaccines. It shows decline in incidence rates of conjunctival papillomas from 2008 to 2025. Vertical lines: Light pink: Female HPV vaccination, Dark pink: Male HPV vaccination, Thick lines: Offical national implementation.1: First catch up programme, girls aged 13–15 years. 2: Implementation of national HPV vaccination for 12‐year‐old girls. 3: Second catch up programme, women aged up to 27 years. 4: Implementation of national HPV vaccination for boys born after 1/7–2007. 5: Two catch up programmes. One included boys born from 1/1–2006 to 30/62007, the other included young men born between 1/11994 and 31/122003 who were attracted to other men.

We included 134 patients, 41.8% (*n* = 56) females and 58.2% (*n* = 78) males. We found a statistically significant decline in incidence over time from 2008 to 2024. The regression coefficient is −0.055 (SE = 0.018, z = −3.06, *p* = 0.002). Expressed as the annual percent change (APC), the incidence declined by −5.3% per year (95% CI: −8.6 to −1.9). This is equivalent to a rate ratio (RR) of 0.95 (95% CI: 0.91–0.98). A graphical representation of the incidence rates over time, with vertical reference lines indicating the introduction of the HPV vaccination for girls and boys including catch‐up programmes, demonstrates a decreasing trend (Figure 5).

Among the 134 patients, 22 had received the HPV vaccine prior to the date of their first biopsy. Of these, 19 had completed 2 or 3 doses. Two patients received the 9‐valent vaccine, while the remaining 20 received the 4‐valent. The interval between the first vaccine dose and the biopsy ranged from 3 months to 15 years, with a mean interval of 5 years and 11 months.

## 4 Discussion

This nationwide Danish retrospective registry study investigated the effect of HPV vaccination on juvenile laryngeal papillomas and conjunctival papillomas. A previous Danish study reported an overall decline in RRP incidence from 1994 to 2021 [14], but post‐vaccination JoRRP incidence has not previously been examined in Denmark. In our study, we observed a modest decline in the incidence of juvenile laryngeal papillomas, although the reduction was not statistically significant. In contrast, an Australian study reported a significant decline in JoRRP from 0.16 in 2012 to 0.02 per 100,000 in 2016, 9 years post‐vaccine introduction [3], while a large American study observed a reduction from a rate of 2.0 to 0.5 per 100.000 from 2004 to 2013 [13].

A considerable proportion of the juvenile laryngeal papilloma patients in our study were diagnosed at a young age, including six patients diagnosed in infancy (0 years of age). The reason for this high proportion of early diagnoses is uncertain, but it may reflect our robust, public health care system. In Denmark, children are examined by a general practitioner at 5 weeks, 5 months and 12 months of age and receive approximately five home visits from a health visitor during their first year of life [15, 16]. This likely facilitates early detection of disease. It should be noted, however, that other studies have included recurrent cases, whereas we report age at first biopsy; therefore, direct comparison of patient ages across studies may not be appropriate.

The first girls included in the Danish childhood vaccination programme are now approximately 28 years old. With the average age for first‐time mothers in Denmark around 30 years [17], we anticipate a further decline in incidence rates as more childbearing women are vaccinated. We found that new cases of juvenile laryngeal papillomas in Denmark declined from five in 2005 to zero in 2024, although only 33 cases were identified across the entire period. With ~1.1 million individuals < 18 years in Denmark, it is difficult to determine whether the observed decline reflects random variation. Across the entire study period, all identified cases of juvenile laryngeal papillomas occurred in children born to unvaccinated mothers, with no cases observed among children of vaccinated mothers, supporting a protective effect of HPV vaccination.

In our study, HPV subtype 6 or 11 was found in ~74% of the biopsies, lower than the > 90% typically reported [2, 18]. Similar to findings from an American study [18], we also identified subtype 70 and a combined subtype as well as HPV negative cases. The VisionArray method used for HPV testing has very high sensitivity and specificity (100% and 96.3%) [19]. Unexpectedly, more than 1/6 of the laryngeal biopsies tested negative for HPV DNA despite histologically confirmed papillomatous tissue, likely due to DNA degradation in older samples or infection with HPV subtypes not included in the testing panel. Notably, four out of five negative biopsies were over 14 years old.

No prior research has examined the impact of HPV vaccine implementation on conjunctival papillomas. We found a significant decline in the incidence of conjunctival papillomas among individuals aged 0–40 years from 2008 to 2024. This decline is likely attributed to the introduction of the HPV vaccination in Denmark. The observed significant reduction in conjunctival papillomas, but not in juvenile laryngeal papillomas, may be explained by a larger number of cases as well as a possible longer latency before vaccine effects can be observed in juvenile laryngeal disease, as reductions depend on vaccinated mothers subsequently influencing disease occurrence in their children. A total of 22 patients had received HPV vaccination prior to their first conjunctival papilloma biopsy. These cases may reflect infection with HPV subtypes not covered by the vaccine or infection acquired prior to vaccination, with vaccination occurring during the incubation period.

### 4.1 Methodological Considerations / Limitations

A strength of this registry‐based study with HPV subtyping is the use of SNOMED codes, which have a high positive predictive value for identifying RRP (98%) [14], exceeding that of ICD‐10 codes [14]. Together with Denmark's comprehensive public health system, this supports that the majority of patients with juvenile laryngeal papillomas were identified.

Limitations include that repeated biopsies are not always performed, why recurrent and single‐occurrence disease could not be distinguished in our dataset. This reduces the risk of underestimating patients with JoRRP but limits comparability with other studies. The statistical power of 18% corresponds to an 82% risk of a type II error, indicating a risk of not detecting a true difference in incidence between the two time periods, even if such a difference exists. This means that the non‐significant findings for juvenile laryngeal papillomas should be interpreted with caution. However, given the rarity of the condition, low statistical power is expected.

Conjunctival papillomas may be underrepresented, as biopsies are not consistently performed in Denmark, and some conjunctival papillomas are removed in private clinics without pathological registration.

### 4.2 Future Directions

Continued surveillance is needed to assess vaccine effects, particularly for JoRRP, where reductions are expected with a delay as vaccinated cohorts reach childbearing age. Expanding the study to other Nordic countries could increase statistical power and allow comparison across similar healthcare systems. Further investigation of the 22 conjunctival papilloma cases despite vaccination may clarify the role of non‐vaccine related HPV types, individual variation in vaccine protection, or infection prior to vaccination.

## 5 Conclusion

In Denmark, we found a non‐significant decline in the incidence of juvenile laryngeal papillomas from 2005 to 2024; however, with no cases recorded in 2024. Of the 33 cases identified, HPV subtype was known for 27 patients, and of these, 74% were attributable to HPV 6 or 11. Mothers were identified for 31 of the laryngeal papilloma patients, and none of the 31 patients' mothers had been vaccinated prior to delivery. In contrast, conjunctival papillomas showed a statistically significant decrease in incidence from 2008 to 2024, corresponding to an annual decline of 5.3%. Collectively, these findings indicate that HPV vaccination has contributed to a reduction in disease burden in Denmark. However, a subset of conjunctival papilloma cases occurred despite prior vaccination, which highlights the need for continued surveillance to monitor long‐term vaccine impact and to investigate cases occurring post‐vaccination.

## Funding

This work was supported by Frimodt‐Heineke Fonden.

## Ethics Statement

The Danish Health Research Ethics Committee system and Team for Medical Records Data approved this study. The General Data Protection Regulation (GDPR) and the Danish Data Protection Act are complied with. Before biopsy retrieval, the biobank manager reviewed the Tissue Utilisation Register to verify whether the patients had declined consent for research. HPV subtyping was conducted without prior written consent. Patients and their families were not informed of the subtyping result as the HPV type does not impact treatment protocols.

## Conflicts of Interest

The authors declare no conflicts of interest.

## Data Availability

Background population counts and statistics on HPV vaccination in Denmark are available at Statistics Denmark [20]. HPV vision array analysis results and the anonymised dataset on papilloma biopsies can be provided upon request. Data regarding vaccination status cannot be disclosed due to national legislation.
